# Combinatorial engineering of hybrid mevalonate pathways in *Escherichiacoli* for protoilludene production

**DOI:** 10.1186/s12934-016-0409-7

**Published:** 2016-01-19

**Authors:** Liyang Yang, Chonglong Wang, Jia Zhou, Seon-Won Kim

**Affiliations:** Division of Applied Life Science (BK21 Plus Program), PMBBRC, Gyeongsang National University, Jinju, 660-701 Korea; Faculty of Life Science and Food Engineering, Huaiyin Institute of Technology, Huai’an, 223003 The People’s Republic of China

**Keywords:** Protoilludene, *Escherichiacoli*, Mevalonate pathway, Sequential order permutation, Homolog substitution

## Abstract

**Background:**

Protoilludene is a valuable sesquiterpene and serves as a precursor for several medicinal compounds and antimicrobial chemicals. It can be synthesized by heterologous expression of protoilludene synthase in *Escherichiacoli* with overexpression of mevalonate (MVA) or methylerythritol-phosphate (MEP) pathway, and farnesyl diphosphate (FPP) synthase. Here, we present *E. coli* as a cell factory for protoilludene production.

**Results:**

Protoilludene was successfully produced in *E. coli* by overexpression of a hybrid exogenous MVA pathway, endogenous FPP synthase (IspA), and protoilludene synthase (OMP7) of *Omphalotusolearius*. For improving protoilludene production, the MVA pathway was engineered to increase synthesis of building blocks isopentenyl diphosphate (IPP) and dimethylallyl diphosphate (DMAPP) by sequential order permutation of the lower MVA portion (MvL), the alteration of promoters and copy numbers for the upper MVA portion (MvU), and the coordination of both portions, resulting in an efficient entire MVA pathway. To reduce the accumulation of mevalonate observed in the culture broth due to lower efficiency of the MvL than the MvU, the MvL was further engineered by homolog substitution with the corresponding genes from *Staphylococcusaureus*. Finally, the highest protoilludene production of 1199 mg/L was obtained from recombinant *E*. *coli* harboring the optimized hybrid MVA pathway in a test tube culture.

**Conclusions:**

This is the first report of microbial synthesis of protoilludene by using an engineered *E. coli* strain. The protoilludene production was increased by approx. Thousandfold from an initial titer of 1.14 mg/L. The strategies of both the sequential order permutation and homolog substitution could provide a new perspective of engineering MVA pathway, and be applied to optimization of other metabolic pathways.

**Electronic supplementary material:**

The online version of this article (doi:10.1186/s12934-016-0409-7) contains supplementary material, which is available to authorized users.

## Background

Protoilludene derivatives, including illudins, marasmanes and melleolides, are known to exert antitumor and antimicrobial activities [1–3]. For example, the most brilliant potential anticancer agent illudin S, which is first isolated from *Omphalotusolearius* mushroom, has been studied extensively owing to its cytotoxicity to various tumor cell types [4]. These biological properties and medicinal potential have attracted considerable attention since the late 1960s. Illudins, marasmanes and melleolides can be synthesized from protoilludene by different oxygenation reactions. For example, P450 monooxygenases for the biosynthesis of illudin have been identified from *O. olearius* [5]. However, protoilludene is naturally produced in a small quantity and its purification from biological material suffers from low yields. Hence, metabolic engineering of microorganisms, such as *Escherichiacoli*, is an alternative and attractive route for the production of protoilludene.

Protoilludene biosynthesis begins with the formation of the universal precursors, isopentenyl pyrophosphate (IPP) and dimethylallyl pyrophosphate (DMAPP), which can be generated via the methylerythritol-phosphate (MEP) pathway and the mevalonate (MVA) pathway [6]. Isopentenyl pyrophosphate and dimethylallyl pyrophosphate are condensed to form farnesyl diphosphate (FPP) by FPP synthase. Linear FPP undergoes multiple electrophilic cyclizations and rearrangements to generate tricyclic protoilludene with an action of protoilludene synthase, which has been isolated from various species including *O.olearius*, *Armillariagallica*, and *Stereumhirsutum* [7–9]. *O.olearius* protoilludene synthase (OMP7) exhibits a superior catalytic efficiency (K_cat_/K_m_) of (13.0 ± 2.0) × 10^4^ M^−1^ s^−1^ among those protoilludene synthases (Additional file 1: Table S1) [8].

**Fig. 1:**
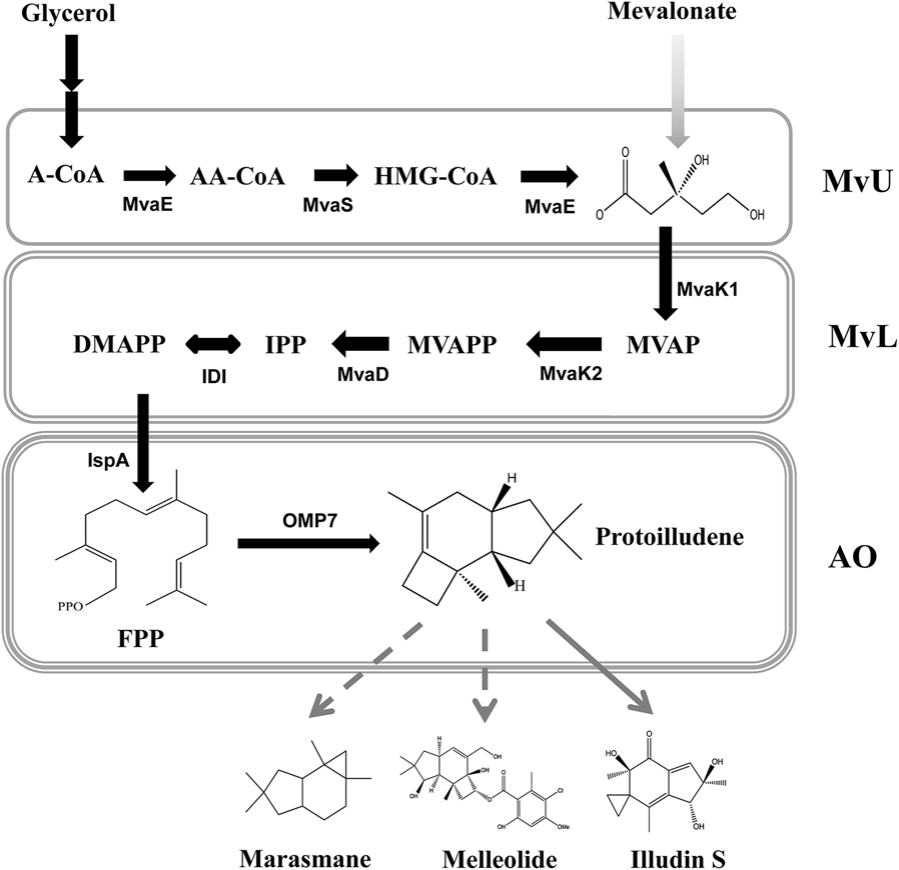
Schematic diagram of protoilludene biosynthesis via mevalonate (MVA) pathway. Protoilludene biosynthesis pathway is divided into three portions: MvU (acetyl-CoA to mevalonate), MvL (mevalonate to IPP and DMAPP) and AO (IPP and DMAPP to protoilludene). MvU portion is composed of MvaE (HMG-CoA reductase/acetyl-CoA acetyltransferase) and MvaS (HMG-CoA synthase). MvL portion is comprised of MvaK1 (mevalonate kinase), MvaK2 (phosphomevalonate kinase), MvaD (diphosphomevalonate decarboxylase) and IDI (IPP isomerase). AO portion consists of IspA (FPP synthase) and OMP7 (protoilludene synthase). Illudins, marasmanes and melleolides are protoilludene derivatives. Pathway intermediates for protoilludene synthesis are as follows: A-CoA, acetyl-CoA; AA-CoA, acetoacetyl-CoA; HMG-CoA, hydroxymethylglutaryl-CoA; MVAP, phosphomevalonate; MVAPP, diphosphomevalonate; IPP, isopentenyl diphosphate; DMAPP, dimethylallyl diphosphate; and FPP, farnesyl diphosphate. *Solid and dashed arrows* indicate the identified and unidentified reactions, respectively

The entire protoilludene synthesis pathway via the MVA pathway can be divided into three portions, referred to as “MvU” composed of acetyl-CoA acetyltransferase/3-hydroxy-3-methylglutaryl-CoA reductase (MvaE) and 3-hydroxy-3-methylglutaryl-CoA synthase (MvaS), “MvL” composed of mevalonate kinase (MvaK1), phosphomevalonate kinase (MvaK2), diphosphomevalonate decarboxylase (MvaD) and IPP isomerase (IDI), and “AO” composed of FPP synthase (IspA) and protoilludene synthase (OMP7) (Fig. 1). The MVA pathway has been widely engineered for production of several sesquiterpenes in *E. coli* [6, 10–12]. In this study, MVA pathway was engineered for a balanced expression of MvU and MvL portions to increase protoilludene production. The MvL portion was optimized by sequential permutation of its constituent genes in consideration of transcriptional polarity, a general tendency of lower expression of the genes distant from promoter in a multi-cistronic operon [13]. In the optimized MvL portion by the random sequential permutation, the constituent genes would be arranged in their activities from low to high activities in the operon. The expression of MvU portion was modulated by changes of promoters and copy-numbers to tune mevalonate production to its utilization by MvL portion. Optimal coordination of the MvUs and MvLs portions of the MVA pathway were finally able to increase protoilludene production from 1.14 to 721 mg/L. As accumulation of mevalonate intermediate was observed in the culture broth, MvL portion was further engineered by substituting its constituent genes with their homologues from *Staphylococcusaureus*. By the homolog substitution, protoilludene production was increased from 721 to 1199 mg/L in a test tube culture. The successful production of protoilludene from *E. coli* is shown in this work and the recombinant *E. coli* harboring the combinatorially engineered hybrid MVA pathway can serve as a basic platform host for production of other valuable terpenoids.

**Table 1:** Strains used in this study

Names	Descriptions	Sources
*E*. *coli* AO	*E*. *coli* DH5α harboring pTAO	This study
*E*. *coli* AO/NA	*E*. *coli* DH5α harboring pTAO and pSNA	This study
*E. coli* AO/MvL_1–6_	*E. coli* DH5α harboring pTAO and pSMvL_1–6_	This study
*E. coli* AO/L1–L6	*E. coli* DH5α harboring pTAO, pSMvL_1–6_ and pBMvU_L_	This study
*E. coli* AO/M1–M13	*E. coli* DH5α harboring pTAO and pSMvL_1–13_-MvU_M_	This study
*E. coli* AO/H1–H13	*E. coli* DH5α harboring pTAOMvU_H_ and pSMvL_1–13_	This study

This table is a brief description of strains used in this study. For more detailed information, refer to Additional file 1: Table S4

## Results and discussion

### Establishment of a protoilludene biosynthesis pathway in *E*. *coli*

**Fig. 2:**
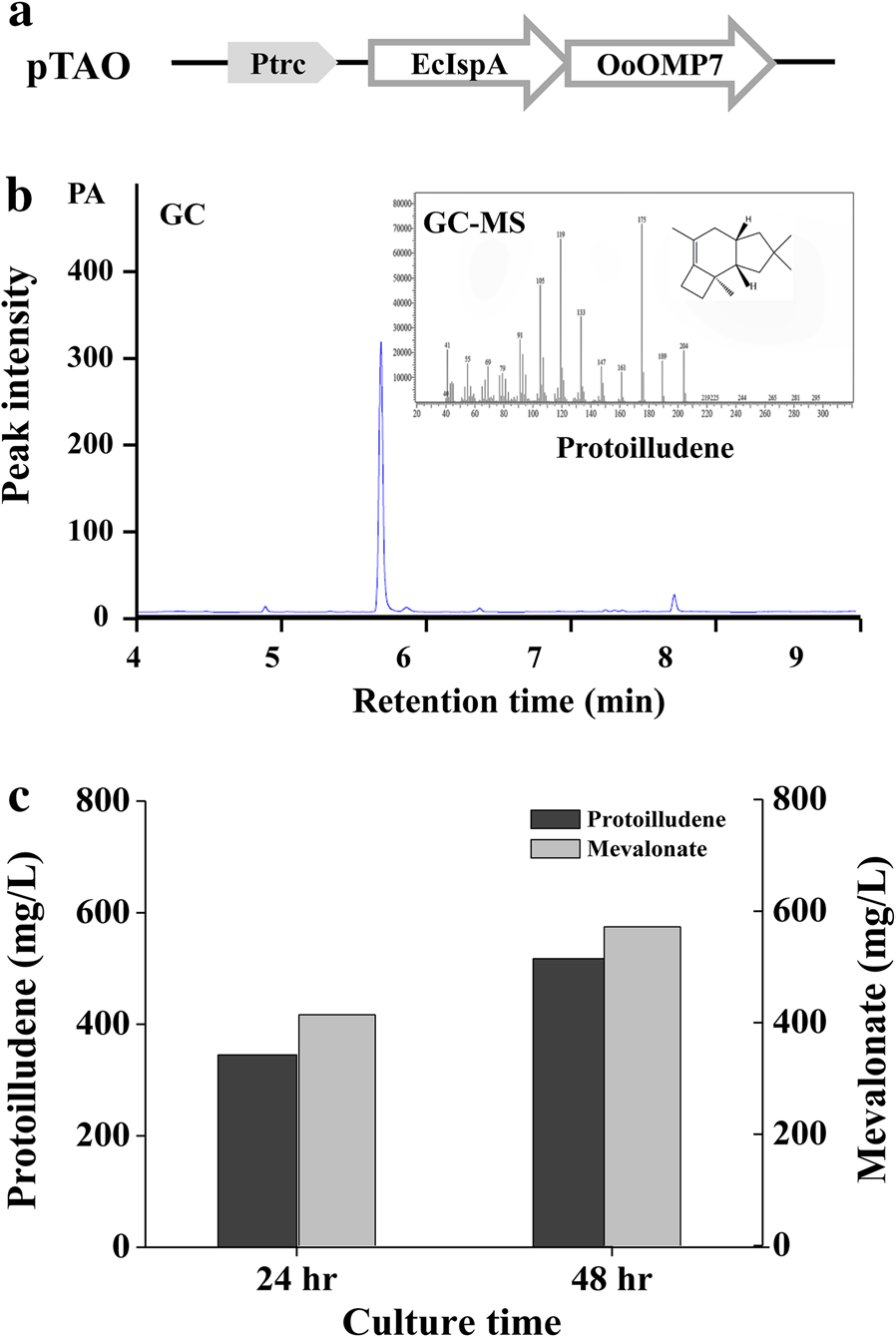
Establishment of a protoilludene biosynthesis pathway in *E*. *coli* **a** Expression construct of AO portion. AO operon, which is composed of FPP synthase *ispA* from *E*. *coli* and protoilludene synthase *OMP7* from *O*. *olearius*, is cloned into pTrc99A vector and designated as pTAO. Ec, the gene from *E. coli*; Oo, the gene from *O. olearius*. **b** Identification of protoilludene product. The decane phase from two-phase culture of *E. coli* AO/NA strain was subjected to GC and GC–MS analysis. **c** Protoilludene production and mevalonate accumulation in culture of the strain *E. coli* AO/NA. The strain was grown at 30 °C in 4 mL of 2YT medium with 2 % (v/v) glycerol for 24 and 48 h, and overlaid with 1 mL of decane. The *dark gray* and *light gray* *bars* indicate protoilludene production and mevalonate accumulation, respectively

Up to now, 6 protoilludene synthases from three species were identified (Additional file 1: Table S1) [7–9]. Among them, *O.olearius* protoilludene synthase (OMP7) exhibits the highest catalytic efficiency which is higher than its homologs OMP6 and Stehi1|73029 by 10 and 30 times, respectively. In order to synthesize protoilludene in *E*. *coli*, a codon-optimized *OMP*7 gene was assembled with *E. coli* FPP synthase gene (*ispA*) to construct plasmid pTAO (Fig. 2a). It was transformed into *E*. *coli* DH5α, resulting in the strain *E*. *coli* AO. This strain was then cultivated at 30 °C for 48 h in 2YT medium containing 2 % (v/v) of glycerol with overlaying 1 mL decane. Gas chromatographic (GC) analysis showed a new peak which was identified as protoilludene by gas chromatograph–mass spectrometer (GC–MS), and corresponded to 1.14 mg/L of protoilludene. The tiny production could be ascribed to an insufficient supply of IPP and DMAPP from the native MEP pathway.

Thus, the protoilludene synthesis plasmid pTAO was co-transformed with plasmid pSNA [14], which encodes a hybrid exogenous MVA pathway for sufficient supply of IPP and DMAPP, into *E. coli* DH5α, resulting in the strain *E. coli* AO/NA. Gas chromatographic analysis showed a specific peak with retention time of 5.7 min, which was subsequently confirmed as protoilludene by GC–MS (Fig. 2b). For 48 h of culture, the strain *E. coli* AO/NA produced 517 mg/L of protoilludene with an undesired accumulation of mevalonate as much as 571 mg/L (Fig. 2c), indicating the suboptimal performance of MVA pathway encoded by pSNA. It is thus required to redesign the MVA pathway, especially the lower portion of the MVA pathway for protoilludene production.

### Optimization of the MvL portion of the MVA pathway by sequential order permutation

**Fig. 3:**
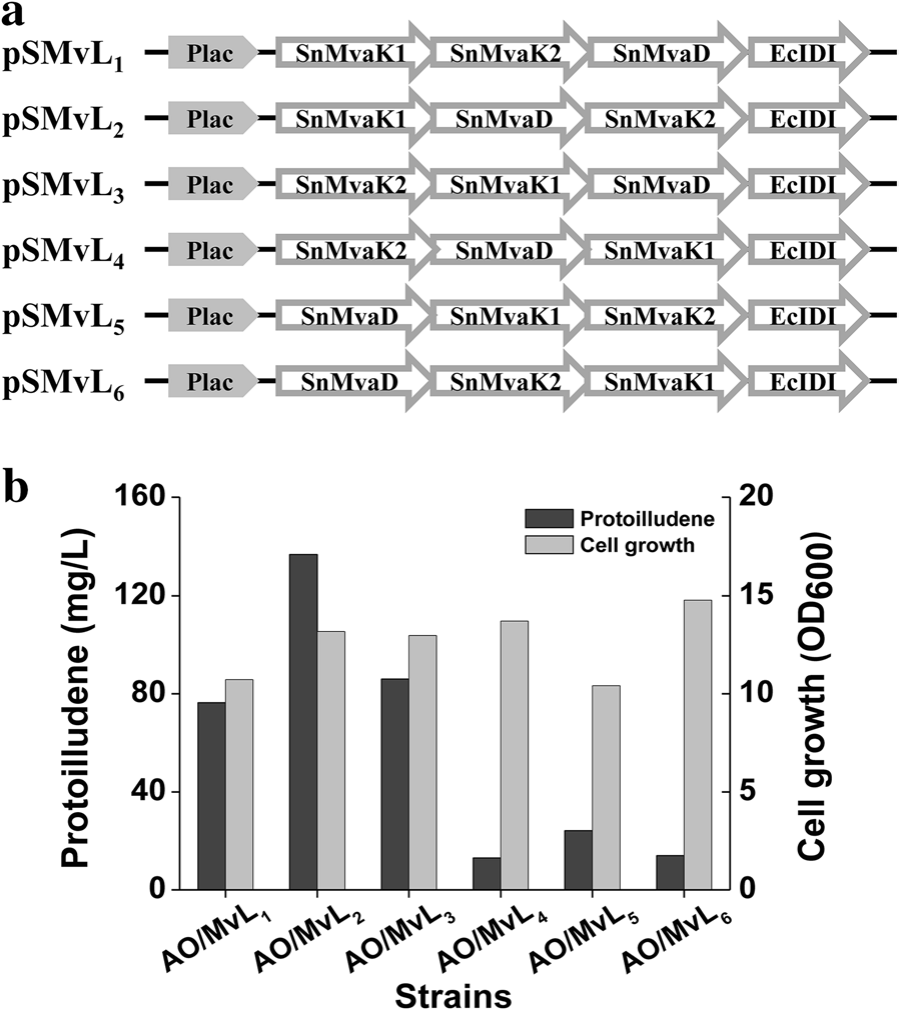
Optimization of the lower (MvL) portion of MVA pathway by sequential order permutation. **a** Expression constructs of the sequential order permutated MvL portions. MvL operon, containing *MvaK1*, *MvaK2* and *MvaD* from *S. pneumoniae*, and *IDI* from *E. coli*, is cloned into pSTV28 vector. Sn, the gene from *S. pneumoniae*; Ec, the gene from *E. coli*; Plac, lac promoter. **b** Effect of sequential order permutated MvLs on protoilludene production and cell growth. The strains were grown for 48 h at 30 °C in 4 mL of 2YT medium containing 2 % (v/v) glycerol and 4 mM mevalonate with overlay of 1 mL decane. The *dark* and *light gray bars* indicate protoilludene production and cell growth, respectively

Expression levels of genes in an operon are known to be affected by their position within the operon [13]. If a gene is located at the tail end of the operon, its expression level is generally lower. Thus, relative expression levels of multi-genes in an operon can be affected by the sequential order of genes in the operon. A specific metabolic pathway encoded by an operon can be optimized by positional modulation of the constituent genes in the operon. Such an approach has been successfully applied to optimization of zeaxanthin synthetic pathway in *Bacillussubtilis* [15]. The MvL portion of pSNA is composed of four genes *SnMvaK1*, *SnMvaK2* and *SnMvaD* from *Streptococcus pneumoniae*, and *IDI* from *E*. *coli* [10]. Optimization of the MvL portion was performed by sequential order permutation of three genes *SnMvaK1*, *SnMvaK2* and *SnMvaD*. The four genes were assembled in a “Biobrick” [16] fashion to construct six sequential order permutated lower MVA pathway plasmids (pSMvL_1–6_) based on pSTV28 vector (Fig. 3a). The strains *E. coli* AO/MvL_1–6_ resulting from the co-transformation of pSMvL_1–6_ and pTAO were evaluated for protoilludene production with supplementation of 4 mM (592.6 mg/L) (R, S)-mevalonate (Fig. 3b). The protoilludene production varied with the sequential order permutation in the MvLs. The highest protoilludene production of 137 mg/L was obtained from *E. coli* AO/MvL_2_, whereas *E. coli* AO/MvL_4–6_ produced low titers of protoilludene below 25 mg/L. Around 80 mg/L of protoilludene was produced from *E. coli* AO/MvL_1, 3_. Residual amounts of mevalonate in the culture broth were measured at the end of the culture to observe the consumption by the strains harboring these sequential order permutated plasmids (Additional file 1: Fig. S2). As expected, the mevalonate consumption generally corresponded to the protoilludene production. The residual mevalonate concentrations in culture of *E. coli* AO/MvL_4–6_ were as high as 3 mM (438.5 mg/L), whereas the concentrations in *E. coli* AO/MvL_1, 3_ and AO/MvL_2_ were as low as around 1.7 mM (248.5 mg/L) and 1.3 mM (190.0 mg/L), respectively. Therefore, the lower MVA pathway plasmid pSMvL_2_ is found to have an optimized gene order for the best performance of the MvL portion, and its order of *SnMvaK1*-*SnMvaD*-*SnMvaK2*-*IDI* is interestingly consistent with arrangement of the native genes in *S. pneumoniae* (GenBank: AE007317.1).

### Coordination of MvU and MvL portions of MVA pathway for protoilludene production

**Fig. 4:**
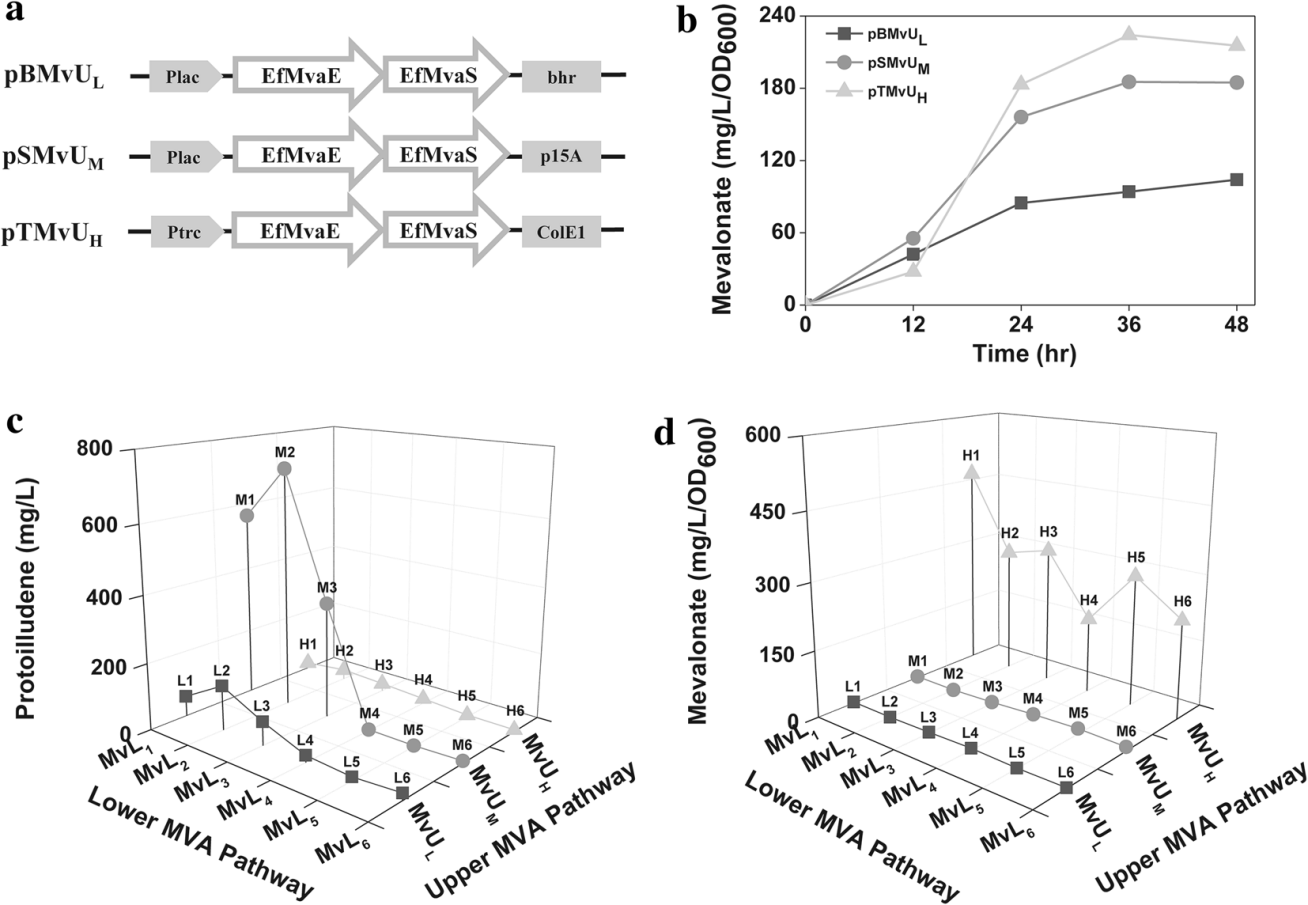
Coordination of the lower (MvL) and upper (MvU) portions of MVA pathway for protoilludene production. **a** Expression constructs of the MvU portions with alternations of promoter and copy-number. MvU operon consists of *MvaE* and *MvaS* from *E. faecalis*. The two genes are cloned into pBBR1MCS-2, pSTV28 and pTrc99A vectors, which are designated as pBMvU_L_, pSMvU_M_ and pTMvU_H_, respectively. Ef, the gene from *E. faecalis*; Plac and Ptrc, lac promoter and trc promoter, respectively. *Rectangles* show replication origin of each plasmid. **b** Mevalonate production capacity of recombinant *E*. *coli* harboring each of pBMvU_L_, pSMvU_M_ and pTMvU_H_. The strains were cultured in 2YT medium at 30 °C for 48 h. **c** and **d** Effect of combinations of MvU_L,M,H_ and MvL_1–6_ on protoilludene production and mevalonate accumulation. The culture were carried out in 4 mL of 2YT medium containing 2 % (v/v) glycerol with overlay of 1 mL decane at 30 °C for 48 h

To optimize the synthesis of mevalonate, the MvU portion of the MVA pathway was cloned into three plasmids with different copy numbers and promoters, pBBR1MCS-2 (6–8 copies and lac promoter), pSTV28 (10–15 copies and lac promoter), and pTrc99A (20–30 copies and trc promoter) [12], which were designated as pBMvU_L_ (LOW), pSMvU_M_ (MEDIUM) and pTMvU_H_ (HIGH), respectively (Fig. 4a). The alternations of copy number and promoter led to the differentiation of mevalonate producing capacity in a range of 104–215 mg/L per OD_600_ at 48 h (Fig. 4b), although there was no significant difference in cell growth among these three strains (Additional file 1: Fig. S3).

Both MvUs and MvLs were then expressed in all combinations in *E. coli* to find an optimal combination of the two portions for protoilludene production. As the MvU_L_ plasmid (pBMvU_L_) is compatible with the lower MVA portion plasmids (pSMvL_1–6_) and the protoilludene plasmid (pTAO), *E. coli* can be transformed with the three plasmids for the combination of MvUs and MvLs in protoilludene production. However, the MvU_M_ plasmid (pSMvU_M_) is not compatible with pSMvL_1–6_ derived from the same cloning vector (pSTV28) and MvU_M_ and MvL_1–6_ are combined in pSMvL_1–6_-MvU_M_ (Additional file 1: Fig. S4). The MvU_H_ portion was cloned into pTAO plasmid, resulting in pTAO-MvU_H_, because the MvU_H_ plasmid (pTMvU_H_) is not compatible with the same vector originated pTAO plasmid (Additional file 1: Fig. S4). *Escherichia coli* AO/H1–H6 strains harboring pTAO-MvU_H_ and pSMvL_1–6_ produced a little amount of protoilludene (<35 mg/L; Fig. 4c), accompanying with accumulation of a large amount of mevalonate (>1300 mg/L). It indicated the MvU_H_ produced too much mevalonate beyond the capacity of MvLs and the metabolic unbalance between MvU_H_ and MvLs caused even a significant decrease of cell growth (Additional file 1: Table S2). In contrast, there was no significant accumulation of mevalonate in the strains of *E. coli* AO/L1-L6 (pTAO/pSMvL_1–6_/pBMvU_L_) and *E. coli* AO/M1–M6 (pTAO/pSMvL_1-6_-MvU_M_), which suggested the lower capacity of the upper portions MvU_L_ and MvU_M_ than the lower portion MvLs (Fig. 4d). In contrast, strains *E. coli* AO/L1–L6 and *E. coli* AO/M1–M6 did not exhibit significant mevalonate accumulation (Fig. 4d). However, the poor mevalonate supply from MvU_L_ compared to MvU_M_ seems to restrict the protoilludene production. The highest protoilludene production of 721 mg/L was observed in *E. coli* AO/M2, which represented a 1.4-fold increase to the production from *E. coli* AO/NA.

### Homolog substitution of the lower MVA portion genes

**Fig. 5:**
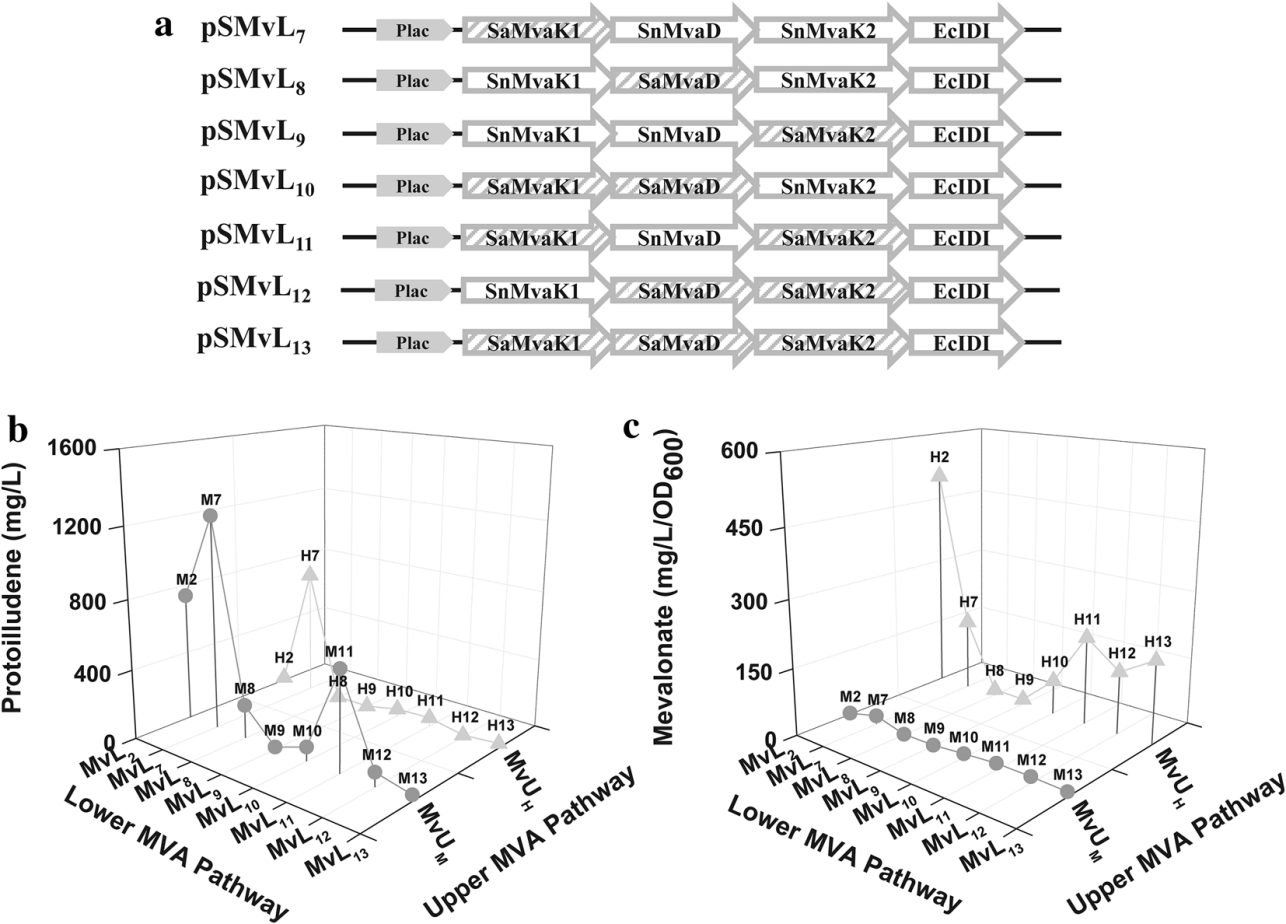
Optimization of MVA pathway by homolog substitution for the *lower* portion genes. **a** Expression constructs of the homolog substituted MvL portions. The homolog genes are from *S. aureus* and represented with prefixion of “Sa” to the gene name. **b** and **c** Effect of combinations of MvU_M,H_ and MvL_7–13_ on protoilludene production and mevalonate accumulation. The culture were carried out in 4 mL of 2YT medium containing 2 % (v/v) glycerol with overlay of 1 mL decane at 30 °C for 48 h

Kinetic properties of homolog enzymes from different organisms are generally distinct from each other. Homolog enzymes of the lower MVA portion have also different kinetic properties. For example, *S. pneumoniae* mevalonate kinase (*SnMvaK1*) is subject to allosteric regulation by diphosphomevalonate, whereas *S. aureus* mevalonate kinase (*SaMvaK1*) without the allosteric regulation is competitively inhibited by isoprenyl diphosphates (DMAPP, IPP and FPP) [17, 18]. A metabolic pathway of interest can be improved by substituting a problematic constituent enzyme with its homolog with a desirable property [14]. In order to further improve the mevalonate pathway, the genes of the lower MVA portion MvL_2_ in pSMvL_2_ were substituted with their homologs from *S. aureus*, resulting into a new set of pSMvL_7–13_ plasmids (Fig. 5a). The effect of the lower MVA portions MvL_7–13_ on protoilludene production was investigated in combination with the upper MVA pathway portions MvU_M_ and MvU_H_ in the same manner used in Fig. 4c. The upper MVA portion MvU_L_ was excluded in this study because it was suspected to produce insufficient amount of mevalonate for high production of protoilludene. The plasmids pSMvL_7–13_-MvU_M_ were constructed to combine the upper portion MvU_M_ and the lower portions MvL_7–13_(Additional file 1: Fig. S4). The combinations of MvU_H_ and MvL_7–13_ were conducted by co-transformation of pTAO-MvU_H_ and pSMvL_7-13_. Interestingly, the strains *E. coli* AO/M7 (pTAO/pSMvL_7_-MvU_M_) and *E. coli* AO/H7 (pTAO-MvU_H_ and pSMvL_7_), containing the lower MVA portion MvL_7_ with homolog substitution of *SaMvaK1* only, produced the enhanced protoilludene production of 1199 and 740 mg/L, respectively (Fig. 5b and Additional file 1: Table S3). Other homolog substitutions failed to improve production of protoilludene (Fig. 5b). As the homolog substitution of *SaMvaK1* with no allosteric inhibition by diphosphomevalonate is effective for protoilludene production, it is suspected the accumulation of diphosphomevalonate in the strain *E*. *coli* AO/M2 harboring the lower MVA portion MvL_2_ due to some bottleneck in the conversion of diphosphomevalonate to IPP by diphosphomevalonate decarboxylase.

## Conclusions

It is demonstrated the feasibility of producing protoilludene in engineered *E. coli*. Heterologous expression of the MVA pathway encoded by pSNA enabled the strain *E. coli* AO/NA to produce 517 mg/L of protoilludene, but mevalonate was accumulated in a significant amount as 571 mg/L due to the unbalanced upper and lower portions of the MVA pathway. To create a balanced efficient MVA pathway, we sequentially permuted the order of genes in the lower portion of the MVA pathway (MvL) and coordinated their expression with the upper portion of the MVA pathway (MvU) by alternations of copy-number and promoter of plasmids. Through this approach, 721 mg/L of protoilludene was produced with reduced accumulation of mevalonate in the strain *E. coli* AO/M2. The substitution of mevalonate kinase from *S. pneumoniae* with the homolog from *S. aureus* further increased protoilludene production to 1199 mg/L. These results suggest that the optimized MVA pathway is efficient to supply IPP and DMAPP for protoilludene production and also can serve as a platform IPP/DMAPP synthesis pathway for production of other valuable terpenes.

## Methods

### Bacterial strains and growth conditions

*Escherichia coli* DH5α were grown in 2YT medium (16 g tryptone, 10 g yeast extract, and 5 g sodium chloride per 1L) at 37 °C for plasmid construction, and at 30 °C for protoilludene production. The seed culture grown overnight at 37 °C was inoculated with an optical density at 600 nm (OD_600_) of 0.1 into 2YT medium containing 2 % (v/v) glycerol. *Escherichia coli* strains  (Table 1) harboring the lower portion of the MVA pathway were cultured with addition of 4 mM mevalonate. Ampicillin (100 μg/mL), chloramphenicol (50 μg/mL), kanamycin (50 μg/mL) and 0.2 mM IPTG were added as required. To harvest protoilludene produced during culture, 1 mL of decane was initially overlaid on 4 mL of culture broth. Cell growth was determined by measuring the OD_600_. All experiments were carried out in duplicate.

### Construction of plasmids

Basic molecular biology procedures, including restriction enzyme digestion and bacterial transformation, were carried out as described in the literature [19]. DNA fragments were amplified by PCR using *Pfu* DNA polymerase (SolGent, Daejeon, Korea) according to the manufacturer’s instructions. BglBricks assembly [16] was applied for construction of various plasmids. The schematic diagram of the constructs is shown in figures and the detailed construction process was depicted in Additional file 1. All plasmids and primers used in this study are described in Additional file 1: Table S4.

### Identification and quantification of protoilludene

The decane phase of the two-phase culture was collected and centrifuged for 10 min at 12,000 rpm to remove cell debris, and subsequently subjected to gas chromatography (GC) and gas chromatography-mass spectrometry (GC–MS). The production of protoilludene was quantified using an Agilent Technologies 7890A gas chromatograph equipped with a flame ionization detector (FID). One μL of sample was injected at a split ratio of 1:10, and separated on a 19091 N-133 HP-INNOWAX column (length, 30 m; internal diameter, 0.25 mm; film thickness, 250 μm). The oven temperature was initially held at 80 °C for 1 min and was increased at a rate of 10 °C/min to 250 °C, where it was held for 1 min. Nitrogen was used as the carrier gas with an inlet pressure of 39 psi. The detector temperature was maintained at 260 °C. GC–MS analysis was run on a GCMS-2010 ultra mass spectrometer (Shimadzu, Tokyo, Japan). Purified protoilludene was used as the standard compound to construct the standard curve (R^2^ > 0.99) for the estimation of protoilludene production (Additional file 1: Fig. S1).

### Quantification of mevalonate

Mevalonate concentration was determined by GC analysis. Culture filtrate was adjusted to pH 2 with 3 M HCl, incubated at 45 °C for 1 h, saturated with anhydrous Na_2_SO_4_, and extracted with ethyl acetate. The resulting samples were analyzed for mevalonate concentration using an Agilent Technologies 7890A gas chromatograph. The analytical temperature of the GC was controlled at an initial temperature of 180 °C for 1 min, then ramped to 200 °C gradually at 2.5 °C/min and held for 2 min. The detector temperature was maintained at 260 °C.

## Additional file

10.1186/s12934-016-0409-7 Construction of plasmids. **Table S1.** Comparison of protoilludene synthases reported in literatures. **Table S2.** Cell growth of recombinant *E. coli* harboring MVA pathway engineered in a way of various combinations of MvU_L,M,H_ and MvL_1–6_. **Table S3.** Cell growth of recombinant *E. coli* harboring MVA pathway engineered with combinations of MvU_M,H_ and MvL_2_,_7–13_. **Table S4.** Strains, plasmids and primers used in this study. **Figure S1.** GC-FID standard curve of protoilludene. **Figure S2.** Residual mevalonate in culture of the strains *E. coli* AO/MvL_1-6_ with exogenous addition of mevalonate. **Figure S3.** Cell growth of *E. coli* strains harboring pBMvU_L_, pSMvU_M_ and pTMvU_H_. **Figure S4.** Schematic diagram of pSMvL_1–13_-MvU_M_ and pTAOMvU_H_.
