# Supplementary material for: Combinatorial engineering of hybrid mevalonate pathways in Escherichiacoli for protoilludene production
Source: Microb Cell Fact. 2016 Jan 19;15:14. doi: 10.1186/s12934-016-0409-7 (PMC4719686; doi:10.1186/s12934-016-0409-7)
Supplement: Supplementary file 1 — 10.1186/s12934-016-0409-7 Construction of plasmids. Table S1. Comparison of protoilludene synthases reported in literatures. Table S2. Cell growth of recombinant E. coli harboring MVA pathway engineered in a way of various combinations of MvUL,M,H and MvL1–6. Table S3. Cell growth of recombinant E. coli harboring MVA pathway engineered with combinations of MvUM,H and MvL2,7–13. Table S4. Strains, plasmids and primers used in this study. Figure S1. GC-FID standard curve of protoilludene. Figure S2. Residual mevalonate in culture of the strains E. coli AO/MvL1-6 with exogenous addition of mevalonate. Figure S3. Cell growth of E. coli strains harboring pBMvUL, pSMvUM and pTMvUH. Figure S4. Schematic diagram of pSMvL1–13-MvUM and pTAOMvUH. [file 12934_2016_409_MOESM1_ESM.docx]

Additional file 1

**Combinatorial engineering of hybrid mevalonate pathways in *Escherichia* *coli* for protoilludene production**

Liyang Yang^a,1^, Chonglong Wang^a,1^, Jia Zhou^a, b^, and Seon-Won Kim^a,*^

^a^ *Division of Applied Life Science (BK21 Plus)*, *PMBBRC*, *Gyeongsang National University*, *Jinju 660-701*, *Korea*

^b^ *Faculty of Life Science and Food Engineering*, *Huaiyin* *Institute of Technology*, *Huai'an 223003, The People 's Republic of China*

^*^ Corresponding author. *E-mail*: swkim@gnu.ac.kr (S.-W. Kim).

Tel.: +82 55 772 1362, Fax: +82 55 759 9363.

*E-mail addresses*: yangyang8028121@hotmail.com (L. Yang).

suzhouwangchonglong@hotmail.com (C. Wang).

jiazhou@hyit.edu.cn (J. Zhou).

^1^ These authors contributed equally to this work.

**Construction of plasmids**

***Construction of protoilludene biosynthesis plasmid***

An artificial protoilludene synthase gene *OMP7* was synthesized by GenScript Corp. (NJ, US), according to *E. coli* codon usage, and inserted into pT-ispA [[1](#_ENREF_1)] digested with BamHI and SalI to produce pTAO (Fig. 2a).

***Construction of lower MVA pathway plasmids by sequential order permutation***

For the construction of pSMvL_1_, mevalonate kinase (*SnMvaK1*) was amplified by using primers SnMvaK1-F with BamHI site and SnMvaK1-R with BglII and SalI sites from the genome of *Streptococcus* *pneumonia* and cloned into pSTV28 digested with BamHI and SalI, resulting in plasmid pS-SnMvaK1. In the same “Biobrick” cloning fashion, phosphomevalonate kinase (*SnMvaK2*) and mevalonate diphosphate decarboxylase (*SnMvaD*) from *S.* *pneumonia*, and IPP isomerase (*EcIDI*) from *E. coli* were amplified by using the primer sets of SnMvaK2-F and SnMvaK2-R, SnMvaD-F and SnMvaD-R, and EcIDI-F and EcIDI-R, respectively, and sequentially cloned into each former plasmid, finally resulting in the lower MVA pathway harboring plasmid pSMvL_1_. For construction of sequentially permutated other lower MVA pathway plasmids, the four fragments, SnMvaK1, SnMvaD, SnMvaK2 and EcIDI were cloned in pSTV28 in different orders, resulting in pSMvL_2_, pSMvL_3_, pSMvL_4_, pSMvL_5_, and pSMvL_6_. The detailed information is presented in the schematic diagram of Fig. 3a.

***Construction of the upper portion of the MVA pathway plasmids with different promoters and copy-numbers***

The upper MVA pathway genes encoding HMG-CoA synthase (*MvaS*) and acetyl-CoA acetyltransferase/HMG-CoA reductase (*MvaE*) were PCR-amplified from plasmid pTEFAES [[2](#_ENREF_2)] by using primers of MvaES-F1 and MvaES-R1. Purified fragment was digested with SalI and BglII and cloned into pBBR1MCS-2 (Plac, 6-8 copies) digested with XhoI and BamHI for construction of pBMvU_L_. In the same manner, PCR fragment, amplified by using primers of MvaES-F2 and MvaES-R2, was digested with BglII and PstI and inserted into pSTV28 (Plac, 10-15 copies) cut with BamHI and PstI to generate pSMvU_M_. For construction of pTMvU_H_, PCR fragment, amplified by using primers of MvaES-F3 and MvaES-R3, was digested with XhoI and PstI and cloned to pTrc99A (Ptrc, 20-30 copies) digested with SalI and PstI. The detailed information was presented in the schematic diagram of Fig. 4a.

For coordination of the upper and lower MVA pathways, the upper MVA pathway genes were PCR-amplified from plasmid pTEFAES [[2](#_ENREF_2)] by using primers of MvaES-F and MvaES-R. The PCR fragment was digested with BglII and XhoI and cloned into pSMvL_1-6_ digested with BglII and SalI to generate pSMvL_1-6_-MvU_M_ (**Additional file 1:** Fig. S4). The same PCR fragment was also digested with with XhoI and PstI and cloned to pTAO digested with SalI and PstI to generate pTAOMvU_H_ (**Additional file 1:** Fig. S4).

***Construction of entire MVA pathway plasmids withhomolog substitution***

Schematic diagram of the lower MVA pathway plasmids with ‘homolog substitution’ is presented in Fig. 5a. As an example, mevalonate kinase from *Staphylococcus* *aureus* (*SaMvaK1*) was PCR-amplified from the genomic DNA of *S*. *aureus* by using primers of SaMvaK1-F with EcoRI site and SaMvaK1-R with BglII and SalI sites. The purified fragment was restricted with EcoRI and SalI and cloned into pSTV28 digested with EcoRI and SalI to create pS-SaMvaK1. The fragments, SnMvaD (primer set: SnMvaD-F/SnMvaD-R, restriction sites: BamHI and BglII/SalI), SnMvaK2 (primer set: SnMvaK2-F/SnMvaK2-R, restriction sites: BamHI and BglII/SalI), EcIDI (primer set: EcIDI-F/EcIDI-R, restriction sites: BamHI and BglII/SalI), were sequentially subcloned into each former plasmids for construction of pSMvL_7_. For the other homolog substitutions, SnMvaK1 (primer set: SnMvaK1-F/SnMvaK1-R, restriction sites: BamHI and BglII/SalI), SaMvaK2 (primer set: SaMvaK2-F/SaMvaK2-R, restriction sites: BamHI and BglII/SalI) and SaMvaD (primer set: SaMvaD-F/SaMvaD-R, restriction sites: BamHI and BglII/SalI) were also used in all combinations to construct other homolog substituted lower MVA pathway plasmids, pSMvL_8-13_by using the aforementioned cloning scheme (Fig. 5a). In order to combine the homolog substituted lower portion MvL_7-13_ with the upper portion MvU_M_, the fragment MvaES (primer set: MvaES-F/MvaES-R, restriction sites: BglII and XhoI) was digested with BglII and XhoI and cloned into pSMvL_7-13_ digested with BglII and SalI, resulting in pSMvL_7-13_-MvU_M_ (**Additional file 1:** Fig. S4).

**Nucleotide sequence of the codon-optimized *OMP7* gene**

ATGCCGGAAACCTTTTATCTGCCGGACTGTCTGGCGAACTGGAAATGGAAACGTGCCCTGAACCCGAACTACCCGGAAGTGAAAGCAGCGAGCTCTGAATGGCTGCGTTCATTTAAAGCCTTCCCGCCGAAAGCACAGGAAGCTTATGATCGCTGCGACTTTAACCTGCTGGCATCGCTGGCATACCCGCTGGCAGATAAAGACGGCCTGCGTACCGGTTGTGATCTGATGAACATGTTTTTCGTTTTCGATGAATACTCAGACGTCGCCCATGAATCGGAAGTCCAGGTGCAAGCGGATATTATCATGGACGCACTGCGTAACCCGCACAAACCGCGTCCGGTCGGTGAATGGGTGGGCGGTGAAGTTACCCGTCAGTTTTGGGAACTGGCGATTAAAACGGCCAGTCCGCAGTCCCAAAAACGCTTTATCGAAACCTTCGATACCTACACGAAAAGCGTGGTTCAGCAAGCGGCCGATCGTACCCAGCATTATGTTCGCACGGTCGATGAATACCTGGAAGTTCGTCGCGACACGATTGGTGCAAAACCGTCTTTCGCTATCCTGGAACTGACCATGGATATCCCGGACGAAGTGATTCATCACCCGACGATCGAACGTCTGGCAATTCTGGCTATCGATATGATTCTGCTGGGCAACGACACCGCATCATATAATTACGAACAGGCTCGCGGTGATGACAACCATAATATGGTGACCATTGTTATGCACCAGTATAAAACGGATATTCAAGGCGCGCTGAGTTGGATCGAAAAATACCACAAAGAACTGGAAGAAGAATTTATGCAGCTGTACAACTCCCTGCCGAAATGGGGCGGTCAAATCGATGTGGATATTGCACGTTATGTGGATGGCCTGGGTAATTGGGTTCGCGCTAGCGATCAGTGGGGCTTTGAATCTGAACGTTACTTCGGTACCAAAGCCCCGGAAATTCAAAAAACCCGCTGGGTGACGCTGATGCCGAAAAAACGTGCCGAAGGTGTTGGCCCGGAAATCGTGGACATCTCAGAACTGTGA

**Table S1.** Comparison of protoilludene synthases reported in literatures.

| **Names** | **Accession No.** | **K_m_[M]*** | **K_cat_/K_m_ [M^-1^s^-1^]*** | **Sources** | **References** |
| --- | --- | --- | --- | --- | --- |
| Pro1 | KC852198 | 0.53 × 10^-6^ | ND | *Armillaria gallica* | B. Engels et al. [[3](#_ENREF_3)] |
| OMP6 | MUStwsD_GLEAN_10003820 | (1.31±0.2) × 10^-5^ | (1.2±0.5) × 10^4^ | *Omphalotus olearius* | G.T. Wawrzyn et al.[[4](#_ENREF_4)] |
| OMP7 | MUStwsD_GLEAN_10000831 | (1.74±0.2) × 10^-6^ | (13.0±2.0) × 10^4^ | *Omphalotus olearius* | G.T. Wawrzyn et al.[[4](#_ENREF_4)] |
| Stehi1Ⅰ25180 | NW_006763134.1 | (5.02±0.9) × 10^-6^ | (8.9±0.7) × 10^2^ | *Stereum hirsutum* | M.B. Quin et al.[[5](#_ENREF_5)] |
| Stehi1Ⅰ64702 | NW_006763145.1 | (1.91±0.3) × 10^-6^ | (19.5±1.5) × 10^2^ | *Stereum hirsutum* | M.B. Quin et al.[[5](#_ENREF_5)] |
| Stehi1Ⅰ73029 | NW_006763132.1 | (1.52±0.2) × 10^-6^ | (41.8±5.1) × 10^2^ | *Stereum hirsutum* | M.B. Quin et al.[[5](#_ENREF_5)] |

*The kinetic properties are obtained by using (*E,E*)-FPP as a substrate in a coupled spectrophotometric assay. "ND" indicates "not determined".

**Table S2.** Cell growth of recombinant *E. coli* harboring MVA pathway engineered in a way of various combinations of MvU_L,M,H_ and MvL_1-6_.

| **Lower**  **Upper** | **MvL_1_** | **MvL_2_** | **MvL_3_** | **MvL_4_** | **MvL_5_** | **MvL_6_** |
| --- | --- | --- | --- | --- | --- | --- |
| **MvU_L_** | 14.1±1.1 | 11.7±1.7 | 8.2±0.9 | 12.4±0.7 | 11.9±0.9 | 10.9±0.4 |
| **MvU_M_** | 20.1±1.4 | 21.4±1.5 | 10.6±0.2 | 6.1±0.3 | 4.1±0.2 | 6.8±0.7 |
| **MvU_H_** | 5.6±0.3 | 6.8±0.1 | 8.5±0.5 | 7.1±0.2 | 4.2±0.2 | 7.6±0.3 |

**Table S3.** Cell growth of recombinant *E. coli* harboring MVA pathway engineered with combinations of MvU_M,H_ and MvL_2_,_7-13_.

| **Lower**  **Upper** | **MvL_2_** | **MvL_7_** | **MvL_8_** | **MvL_9_** | **MvL_10_** | **MvL_11_** | **MvL_12_** | **MvL_13_** |
| --- | --- | --- | --- | --- | --- | --- | --- | --- |
| **MvU_M_** | 21.4±1.5 | 17.0±2.5 | 18.1±0.1 | 24.8±1.7 | 17.3±0.5 | 18.1±0.2 | 11.7±0.9 | 14.5±2.1 |
| **MvU_H_** | 6.7±0.1 | 17.9±0.1 | 10.0±0.6 | 8.5±0.1 | 8.6±0.7 | 8.4±0.2 | 7.2±0.1 | 7.4±0.1 |

**Table S4.** Strains, plasmids and primers used in this study.

| **Names** | **Descriptions** | **References or sources** |
| --- | --- | --- |
| **Strains** |  |  |
| *E*. *coli* DH5α | F^-^, Φ80dlacZDM15, Δ(*lacZYA*-*argF*)U169, *deoR*, *recA*1, *endA*1, *hsdR*17(r_K_^_^ m_K_+), *phoA*, *supE*44, λ^-^, *thi*-1 | ATCC |
| *E*. *coli* AO | *E*. *coli* DH5α harboring pTAO | This study |
| *E*. *coli* AO/NA | *E*. *coli* DH5α harboring pTAO and pSNA | This study |
| *E. coli* AO/MvL_1_ | *E. coli* DH5α harboring pTAO and pSMvL_1_ | This study |
| *E. coli* AO/ MvL_2_ | *E. coli* DH5α harboring pTAO and pSMvL_2_ | This study |
| *E. coli* AO/ MvL_3_ | *E. coli* DH5α harboring pTAO and pSMvL_3_ | This study |
| *E. coli* AO/ MvL_4_ | *E. coli* DH5α harboring pTAO and pSMvL_4_ | This study |
| *E. coli* AO/ MvL_5_ | *E. coli* DH5α harboring pTAO and pSMvL_5_ | This study |
| *E. coli* AO/ MvL_6_ | *E. coli* DH5α harboring pTAO and pSMvL_6_ | This study |
| *E. coli* AO/L1 | *E. coli* DH5α harboring pTAO, pSMvL_1_ and pBMvU_L_ | This study |
| *E. coli* AO/ L2 | *E. coli* DH5α harboring pTAO, pSMvL_2_ and pBMvU_L_ | This study |
| *E. coli* AO/ L3 | *E. coli* DH5α harboring pTAO, pSMvL_3_ and pBMvU_L_ | This study |
| *E. coli* AO/ L4 | *E. coli* DH5α harboring pTAO, pSMvL_4_ and pBMvU_L_ | This study |
| *E. coli* AO/ L5 | *E. coli* DH5α harboring pTAO, pSMvL_5_ and pBMvU_L_ | This study |
| *E. coli* AO/ L6 | *E. coli* DH5α harboring pTAO, pSMvL_6_ and pBMvU_L_ | This study |
| *E. coli* AO/ M1 | *E. coli* DH5α harboring pTAO and pSMvL_1_-MvU_M_ | This study |
| *E. coli* AO/ M2 | *E. coli* DH5α harboring pTAO and pSMvL_2_-MvU_M_ | This study |
| *E. coli* AO/ M3 | *E. coli* DH5α harboring pTAO and pSMvL_3_-MvU_M_ | This study |
| *E. coli* AO/ M4 | *E. coli* DH5α harboring pTAO and pSMvL_4_-MvU_M_ | This study |
| *E. coli* AO/ M5 | *E. coli* DH5α harboring pTAO and pSMvL_5_-MvU_M_ | This study |
| *E. coli* AO/ M6 | *E. coli* DH5α harboring pTAO and pSMvL_6_-MvU_M_ | This study |
| *E. coli* AO/M7 | *E. coli* DH5α harboring pTAO and pSMvL_7_-MvU_M_ | This study |
| *E. coli* AO/M8 | *E. coli* DH5α harboring pTAO and pSMvL_8_-MvU_M_ | This study |
| *E. coli* AO/M9 | *E. coli* DH5α harboring pTAO and pSMvL_9_-MvU_M_ | This study |
| *E. coli* AO/M10 | *E. coli* DH5α harboring pTAO and pSMvL_10_-MvU_M_ | This study |
| *E. coli* AO/M11 | *E. coli* DH5α harboring pTAO and pSMvL_11_-MvU_M_ | This study |
| *E. coli* AO/M12 | *E. coli* DH5α harboring pTAO and pSMvL_12_-MvU_M_ | This study |
| *E. coli* AO/M13 | *E. coli* DH5α harboring pTAO and pSMvL_13_-MvU_M_ | This study |
| *E. coli* AO/H1 | *E. coli* DH5α harboring pTAOMvU_H_ and pSMvL_1_ | This study |
| *E. coli* AO/H2 | *E. coli* DH5α harboring pTAOMvU_H_ and pSMvL_2_ | This study |
| *E. coli* AO/H3 | *E. coli* DH5α harboring pTAOMvU_H_ and pSMvL_3_ | This study |
| *E. coli* AO/H4 | *E. coli* DH5α harboring pTAOMvU_H_ and pSMvL_4_ | This study |
| *E. coli* AO/H5 | *E. coli* DH5α harboring pTAOMvU_H_ and pSMvL_5_ | This study |
| *E. coli* AO/H6 | *E. coli* DH5α harboring pTAOMvU_H_ and pSMvL_6_ | This study |
| *E. coli* AO/H7 | *E. coli* DH5α harboring pTAOMvU_H_ and pSMvL_7_ | This study |
| *E. coli* AO/H8 | *E. coli* DH5α harboring pTAOMvU_H_ and pSMvL_8_ | This study |
| *E. coli* AO/H9 | *E. coli* DH5α harboring pTAOMvU_H_ and pSMvL_9_ | This study |
| *E. coli* AO/H10 | *E. coli* DH5α harboring pTAOMvU_H_ and pSMvL_10_ | This study |
| *E. coli* AO/H11 | *E. coli* DH5α harboring pTAOMvU_H_ and pSMvL_11_ | This study |
| *E. coli* AO/H12 | *E. coli* DH5α harboring pTAOMvU_H_ and pSMvL_12_ | This study |
| *E. coli* AO/H13 | *E. coli* DH5α harboring pTAOMvU_H_ and pSMvL_13_ | This study |
| **Plasmids** |  |  |
| pSTV28 | P_lac_ expression vector, pACYC184 origin, *lacZ*, *Cm*^r^ | Takara Co., Ltd. |
| pTrc99A | Ptrc expression vector, ColE1 origin, *lacI*_q_, *Amp*^r^ | Amann et al. (1988) |
| pBBR1MCS-2 | P_lac_ expression vector, *lacZ*, *Km*^r^ | Kovach et al.(1995) |
| pTispA | pTrc99A vector containing FPP synthase *ispA* from *E*. *coli* | Wang et al. (2010) |
| pTAO | pTrc99A vector containing FPP synthase *ispA* from *E*. *coli* and protoilludene synthase *OMP7* from *O.olearius* | This study |
| pSNA | pSTV28 containing *MvaE* and *MvaS* of *E*. *faecalis*, *MvaK1*, *MvaK2*, and *MvaD* of *S. pneumoniae*, and *IDI* of *E. coli* | Yoon et al. (2009) |
| pSMvL_1_ | pSTV28 vector containing *MvaK1*-*MvaK2*-*MvaD* from *S*. *pneumoniae*, and *IDI* from *E*. *coli* | This study |
| pSMvL_2_ | pSTV28 vector containing *MvaK1*-*MvaD*-*MvaK2* from *S*. *pneumoniae*, and *IDI* from *E*. *coli* | This study |
| pSMvL_3_ | pSTV28 vector containing *MvaK2*-*MvaK1*-*MvaD* from *S*. *pneumoniae*, and *IDI* from *E*. *coli* | This study |
| pSMvL_4_ | pSTV28 vector containing *MvaK2*-*MvaD*-*MvaK1* from *S*. *pneumoniae*, and *IDI* from *E*. *coli* | This study |
| pSMvL_5_ | pSTV28 vector containing *MvaD*-*MvaK1*-*MvaK2* from *S*. *pneumoniae*, and *IDI* from *E*. *coli* | This study |
| pSMvL_6_ | pSTV28 vector containing *MvaD*-*MvaK2*-*MvaK1* from *S*. *pneumoniae*, and *IDI* from *E*. *coli* | This study |
| pSMvL_7_ | pSTV28 vector containing *MvaK1* from *S. aureus*, *MvaD* from *S*. *pneumonia*, *MvaK2* from *S*. *pneumoniae*, and *IDI* from *E*. *coli* | This study |
| pSMvL_8_ | pSTV28 vector containing *MvaK1* from *S*. *pneumonia*, *MvaD* from *S*. *aureus*, *MvaK2* from *S*. *pneumoniae*, and *IDI* from *E*. *coli* | This study |
| pSMvL_9_ | pSTV28 vector containing *MvaK1* from *S*. *pneumonia*, *MvaD* from *S*. *pneumonia*, *MvaK2* from *S*. *aureus* and *IDI* from *E*. *coli* | This study |
| pSMvL_10_ | pSTV28 vector containing *MvaK1* from *S*. *aureus*, *MvaD* from *S*. *aureus*, *MvaK2* from *S*. *pneumoniae*, and *IDI* from *E*. *coli* | This study |
| pSMvL_11_ | pSTV28 vector containing *MvaK1* from *S*. *pneumonia*, *MvaD* from *S*. *aureus*, *MvaK2* from *S*. *aureus* and *IDI* from *E*. *coli* | This study |
| pSMvL_12_ | pSTV28 vector containing *MvaK1* from *S*. *aureus*, *MvaD* from *S*. *pneumonia*, *MvaK2* from *S*. *aureus*, and *IDI* from *E*. *coli* | This study |
| pSMvL_13_ | pSTV28 vector containing *MvaK1*-*MvaD*-*MvaK2* from *S*. *aureus*, and IDI from *E*. *coli* | This study |
| pBMvU_L_ | pBBRmcs-2 vector containing *MvaE* and *MvaS* from *E*. *faecalis* | This study |
| pSMvU_M_ | pSTV28 vector containing *MvaE* and *MvaS* from *E*. *faecalis* | This study |
| pTMvU_H_ | pTrc99A vector containing *MvaE* and *MvaS* from *E*. *faecalis* | This study |
| pTAOMvU_H_ | pTrc99A vector containing *ispA* from *E*.*coli*, protoilludene synthase *OMP7* from *O*.*olearius* and *MvaE* and *MvaS* from *E*. *faecalis* | This study |
| pSMvL_1_-MvU_M_ | pSTV28 vector containing MvL_1_ portion and MvU_M_ portion | This study |
| pSMvL_2_-MvU_M_ | pSTV28 vector containing MvL_2_ portion and MvU_M_ portion | This study |
| pSMvL_3_-MvU_M_ | pSTV28 vector containing MvL_3_ portion and MvU_M_ portion | This study |
| pSMvL_4_-MvU_M_ | pSTV28 vector containing MvL_4_ portion and MvU_M_ portion | This study |
| pSMvL_5_-MvU_M_ | pSTV28 vector containing MvL_5_ portion and MvU_M_ portion | This study |
| pSMvL_6_-MvU_M_ | pSTV28 vector containing MvL_6_ portion and MvU_M_ portion | This study |
| pSMvL_7_-MvU_M_ | pSTV28 vector containing MvL_7_ portion and MvU_M_ portion | This study |
| pSMvL_8_-MvU_M_ | pSTV28 vector containing MvL_8_ portion and MvU_M_ portion | This study |
| pSMvL_9_-MvU_M_ | pSTV28 vector containing MvL_9_ portion and MvU_M_ portion | This study |
| pSMvL_10_-MvU_M_ | pSTV28 vector containing MvL_10_ portion and MvU_M_ portion | This study |
| pSMvL_11_-MvU_M_ | pSTV28 vector containing MvL_11_ portion and MvU_M_ portion | This study |
| pSMvL_12_-MvU_M_ | pSTV28 vector containing MvL_12_ portion and MvU_M_ portion | This study |
| pSMvL_13_-MvU_M_ | pSTV28 vector containing MvL_13_ portion and MvU_M_ portion | This study |
| **Primers** |  |  |
| OMP7-F | AC*GGATCC*AAGGAGATATATCAA**ATG**CCGGAAACCTTTTATCT | This study |
| OMP7-R | TATC*GTCGAC***TCA**CAGTTCTGAGATGTCC | This study |
| SnMvaK1-F | AC*GGATCC*TAAGGAACACAGTTTT**ATG**ACAAAAAAAGTTGGTGTC | This study |
| SnMvaK1-R | TATC*GTCGAC*TCTA*AGATC****T*TA**CAGGCTCTCTATCCATGTC | This study |
| SnMvaD-F | AC*GGATCC*AATAAGGAGGTCAACA**ATG**GATAGAGAGCCTGTAACAG | This study |
| SnMvaD-R | GACT*GTCGAC*TCTA*AGATC****T*TA**ACAGCAATCATCTTGACTC | This study |
| SnMvaK2-F | AC*GGATCC*TACAAGGAGGTACCAA**ATG**ATTGCTGTTAAAACTTGCG | This study |
| SnMvaK2-R | TATC*GTCGAC*TCTA*AGATC****T*TA**CGATTTGTCGTCATGTCCTATC | This study |
| EcIDI-F | AC*GGATCC*TGAGGAGGTAACGT**ATG**CAAACGGAACACGTCATTTTA | This study |
| EcIDI-R | TATC*GTCGAC*TCTA*AGATC****T*TA**TTTAAGCTGGGTAAATGCAG | This study |
| SaMvaK1-F | AC*GAATTC*GAGGGGGGCATCCG**ATG**ACAAGAAAAGGATATGGG | This study |
| SaMvaK1-R | TATC*GTCGAC*TCTA*AGATC****T*TA**ACCTCCTAAATTCTCAATC | This study |
| SaMvaD-F | AC*GGATCC*GAGGAGGTATACTTA**ATG**ATTAAAAGTGGCAAAGCACG | This study |
| SaMvaD-R | GACT*GTCGAC*TCTA*AGATC****T*TA**CTCAATTATTTCAATTCCTG | This study |
| SaMvaK2-F | AC*GGATCC*CAAAGGAGGTCCAAT**ATG**ATTCAGGTCAAAGCACCCG | This study |
| SaMvaK2-R | TATC*GTCGAC*TCTA*AGATC***TTA**TTGCCCATGATAAATATTAAAT | This study |
| MvaES-F | TATC*AGATCT*ACGAGGAGGGTCTATT**ATG**AAAACAGTAGTTATTATTG | This study |
| MvaES-R | TCGA*CTCGAG***TTA**GTTTCGATAAGAGCGAACGG | This study |
| MvaES-F1 | TATC*GTCGAC*ACGAGGAGGGTCTATT**ATG**AAAACAGTAGTTATTATTG | This study |
| MvaES-R1 | TATC*AGATC***TTA**GTTTCGATAAGAGCGAACGG | This study |
| MvaES-F2 | TATC*AGATCT*ACGAGGAGGGTCTATT**ATG**AAAACAGTAGTTATTATTG | This study |
| MvaES-R2 | TCGA*CTCGAG***TTA**GTTTCGATAAGAGCGAACGG | This study |
| MvaES-F3 | TCGA*CTCGAG*ACGAGGAGGGTCTATT**ATG**AAAACAGTAGTTATTATTG | This study |
| MvaES-R3 | TCGA*CTGCAG***TTA**GTTTCGATAAGAGCGAACGG | This study |

*Note*: Oligonucleotide sequences are indicted in the 5’-to-3’ direction. Italic nucleotides indicate restriction sites. The start codons and the stop codons (complementary sequences) of genes are indicated as bold letters.

**
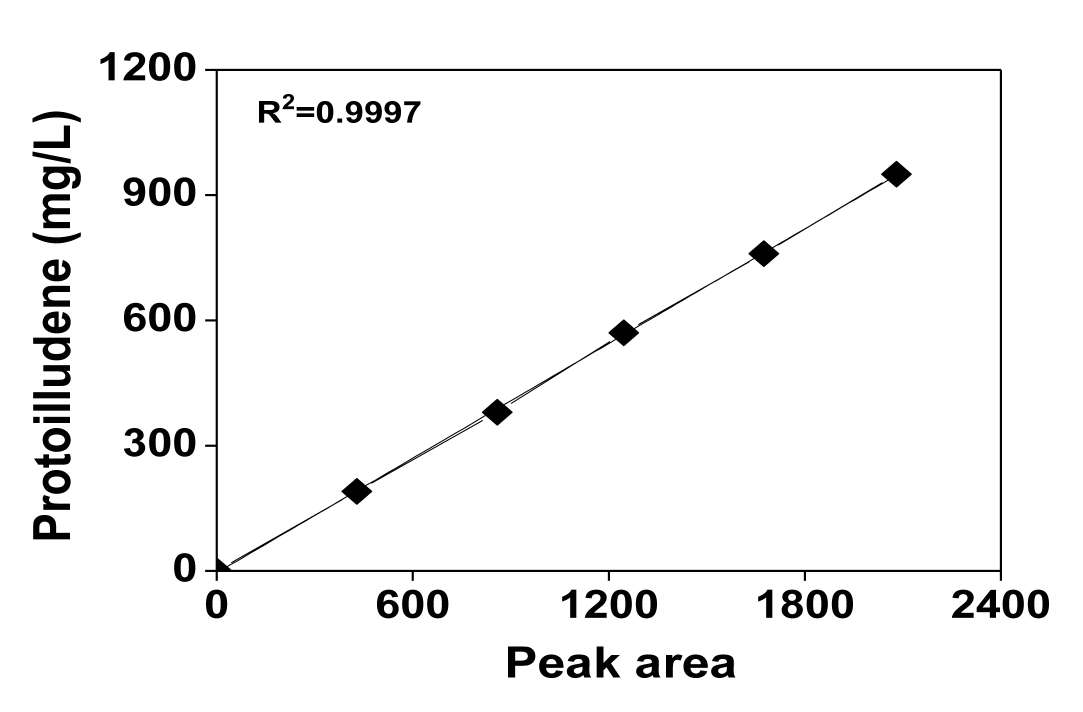
**

**Figure S1. GC-FID standard curve of protoilludene.**

**
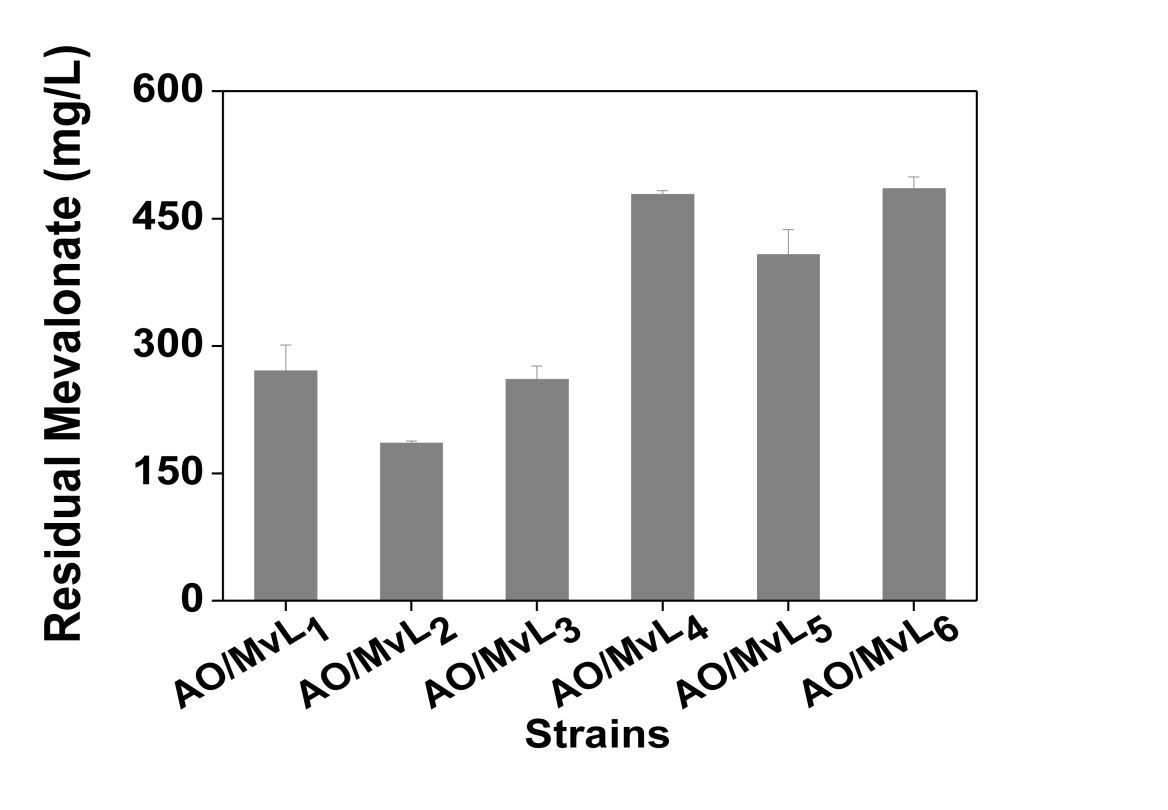
**

**Figure S2. Residual mevalonate in culture of the strains *E. coli* AO/MvL_1-6_ with exogenous addition of mevalonate.** Strains were cultured at 30 ^°^C in 2YT medium containing 4 mM mevalonate and 2.0 % (v/v) glycerol. The residual mevalonate was measured after 48 hours of culture.

**
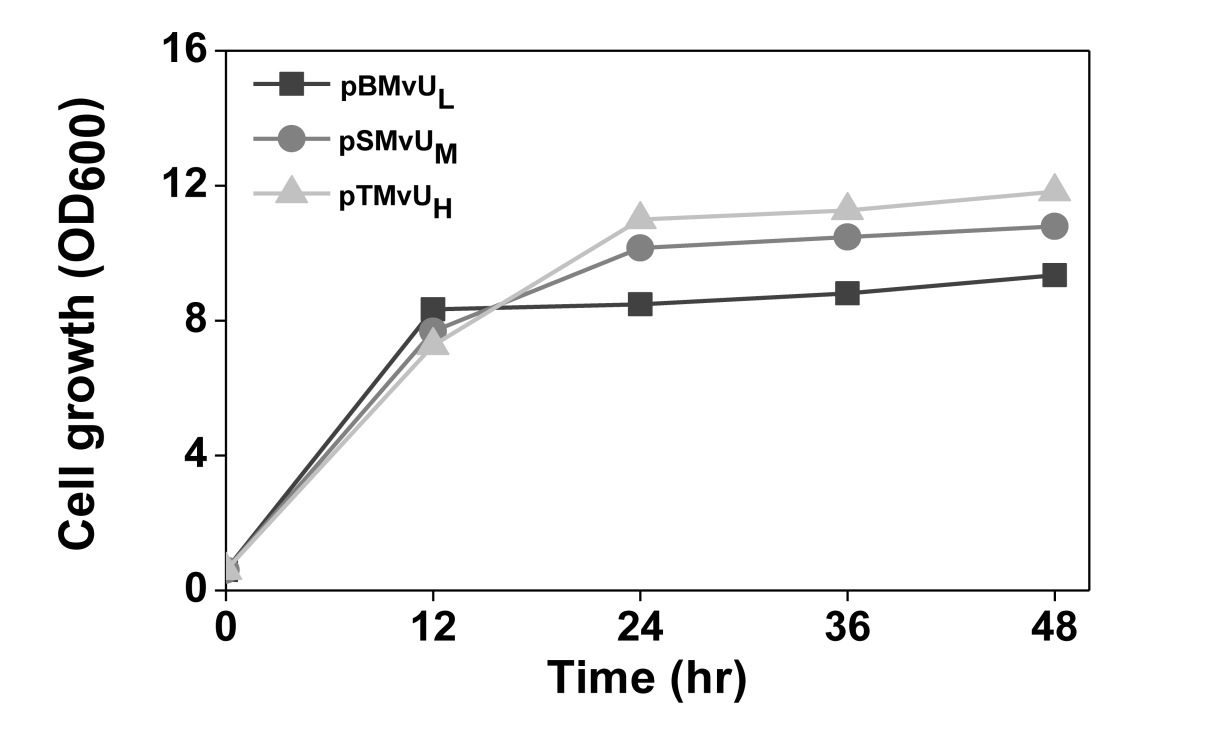
**

**Figure S3. Cell growth of *E.* *coli* strains harboring pBMvU_L_, pSMvU_M_ and pTMvU_H_.** The strains were cultured in 2YT medium at 30 ^°^C for 48 hours.

**
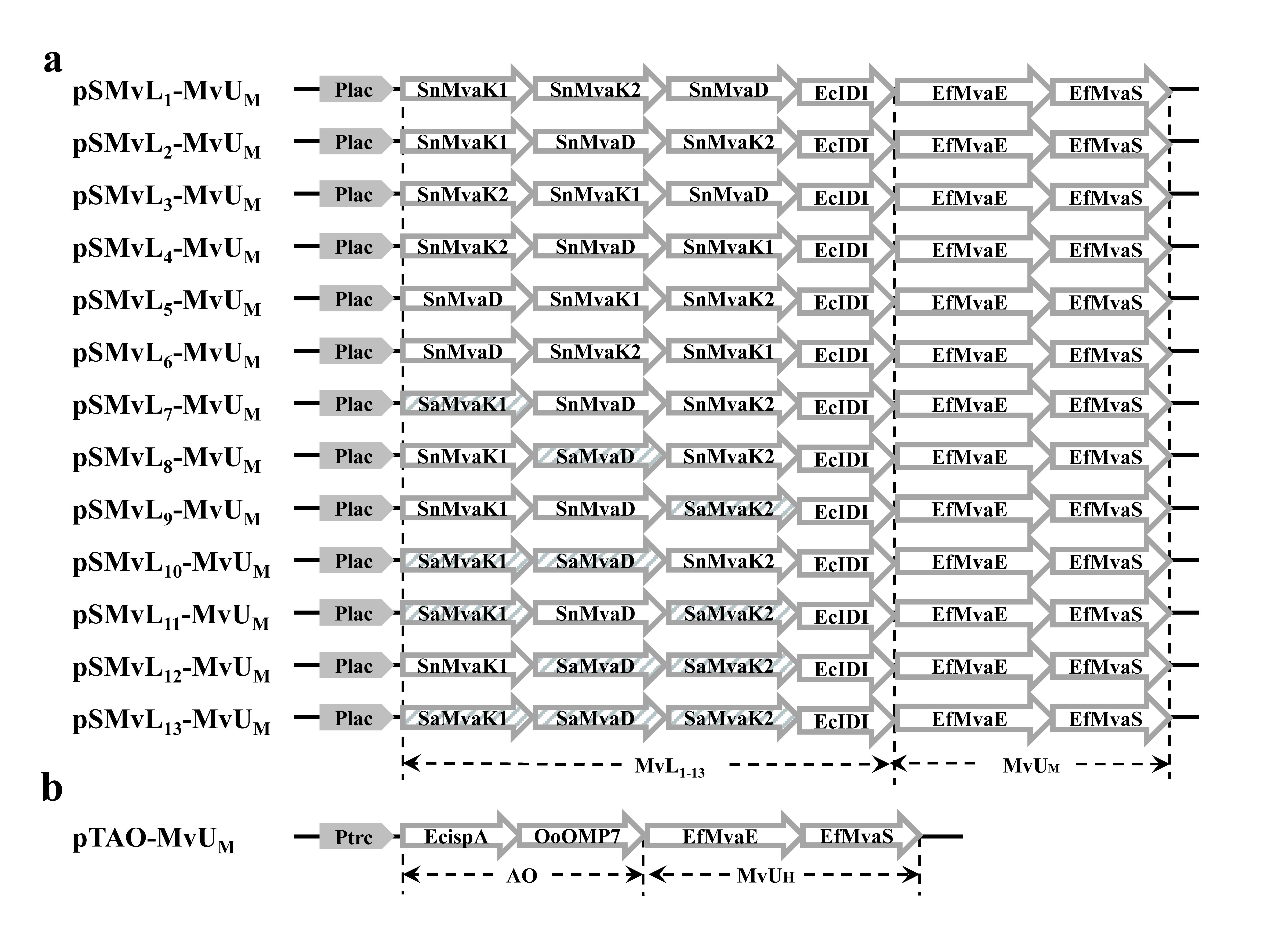
**

**Figure S4. Schematic diagram of pSMvL_1-13_-MvU_M_ and pTAOMvU_H_**. The pentagons and arrows represent promoters and genes, respectively. The “Sn”, “Ec”, “Ef”, and “Oo” indicate the genes from *S*. *pneumonia*, *E*. *coli*, *E*. *faecalis*, and *O*. *olearius*, respectively.

**References**

1. Wang C, Yoon SH, Shah AA, Chung YR, Kim JY, Choi ES, Keasling JD, Kim SW: **Farnesol production from *Escherichia* *coli* by harnessing the exogenous mevalonate pathway.** *Biotechnol Bioeng* 2010, **107:**421-429.

2. Yoon SH, Lee SH, Das A, Ryu HK, Jang HJ, Kim JY, Oh DK, Keasling JD, Kim SW: **Combinatorial expression of bacterial whole mevalonate pathway for the production of beta-carotene in *E*. *coli*.** *J Biotechnol* 2009, **140:**218-226.

3. Engels B, Heinig U, Grothe T, Stadler M, Jennewein S: **Cloning and characterization of an *Armillaria* *gallica* cDNA encoding protoilludene synthase, which catalyzes the first committed step in the synthesis of antimicrobial melleolides.** *J Biol Chem* 2011, **286:**6871-6878.

4. Wawrzyn GT, Quin MB, Choudhary S, Lopez-Gallego F, Schmidt-Dannert C: **Draft genome of *Omphalotus* *olearius* provides a predictive framework for sesquiterpenoid natural product biosynthesis in Basidiomycota.** *Chem Biol* 2012, **19:**772-783.

5. Quin MB, Flynn CM, Wawrzyn GT, Choudhary S, Schmidt-Dannert C: **Mushroom hunting by using bioinformatics: application of a predictive framework facilitates the selective identification of sesquiterpene synthases in Basidiomycota.** *Chembiochem* 2013, **14:**2480-2491.
